# The use of deuterium-labeled *gamma*-aminobutyric (D_6_-GABA) to study uptake, translocation, and metabolism of exogenous GABA in plants

**DOI:** 10.1186/s13007-020-00574-9

**Published:** 2020-02-27

**Authors:** Faraj Hijaz, Nabil Killiny

**Affiliations:** Department of Plant Pathology, Citrus Research and Education Center, IFAS, University of Florida, 700 Experiment Station Road, Lake Alfred, FL 33850 USA

**Keywords:** *Gamma*-aminobutyric acid, 4-aminobutyric acid, γ-aminobutyric acid, GABA, Succinic acid, Translocation

## Abstract

**Background:**

Exogenous application of *gamma*-aminobutyric acid (GABA) could relieve stress symptoms caused by abiotic stresses including anoxia, heat and cold shock, drought, and salt. However, studying translocation and metabolism of exogenous GABA is challenged by the presence of endogenous GABA.

**Results:**

Herein, we used D_6_-GABA in order to investigate the uptake, translocation, and the metabolism of exogenous GABA in Mexican lime (*Citrus aurantifolia*) seedlings using gas chromatography-mass spectrometry (GC–MS). The GC–MS analysis showed that D_6_-GABA could be easily distinguished from the non-labeled GABA after methyl chloroformate (MCF) derivatization. The D_6_-GABA was detected in the cortex (phloem), inner stem (xylem), and leaves after root drench. Girdling did not affect the translocation of D_6_-GABA, indicating that it is mainly translocated via the xylem. In addition, D_4_-labled succinic acid was detected in D_6_-GABA-treated plants, indicating that exogenous GABA was metabolized to succinic acid. The half-life of D_6_-GABA in citrus was about 1.3 h, indicating a quick conversion to succinic acid.

**Conclusion:**

The use of D_6_-GABA offers a valuable tool to study the translocation and metabolism of GABA in plants. D_6_-GABA and its metabolite (D_4_-succinic acid) can be easily distinguished from the endogenous GABA and succinic acid using GC–MS.

## Background

γ-aminobutyric (GABA) is widely distributed in plants and it has received a great attention in plant science [1]. This non-proteinogenic amino acid (NPAA) is synthesized in the cytosol from glutamate by glutamate decarboxylase (GAD) [1]. The level of GABA in plants increases under biotic (e.g. insects and viral attack) and abiotic stresses (e. g. anoxia, heat and cold shock, drought, and salt) [1, 2]. GABA could also be synthesized from putrescine by D-amino oxidase (DAO) and spermidine by polyamine oxidase [3]. Liao et al. (2017) showed that DAO contributed about one-fourth of GABA formed in tea leaves under anoxia [3]. Non-enzymatic degradation of proline under oxidative stress could also contribute to GABA formation [4].

The increase in cytosolic Ca^+2^ and/or H^+^ leads to the activation of GAD [1, 2]. Accumulation of Ca^+2^ occurs under many abiotic stresses including salinity, heat shock, and drought. Accumulation of H^+^ was also reported under various stresses including anoxia and mechanical damage [1, 2]. Accumulation of Ca^+2^ at normal physiological pH results in the formation of Ca^+2^/calmodulin complex leading to the activation of GAD enzyme [1, 2]. Activation of GAD by Ca^+2^/calmodulin complex occurs during mild or early stages of stresses [1, 2]. However, during severe and late stages of stress (at low cytosolic pH) the GAD enzyme is activated in a pH-dependent manner [1, 2].

Previous reports showed that exogenous application of GABA to plants could enhance their growth and relieve stress symptoms caused by abiotic stresses. For example, exogenous application of GABA on *Caragana intermedia* roots decreased H_2_O_2_ levels and increased ethylene production under salt stress [5]. In the same manner, GABA increased the antioxidant enzyme activity and decreased reactive oxygen species (ROS) in muskmelon seedlings under hypoxia stress [6]. Moreover, the levels of several sugars, amino acids, and organic acids was enhanced upon the application of GABA in creeping bentgrass (*Agrostis stolonifera*) under heat stress [7]. Exogenous application of GABA improved drought tolerance and increased the level of several amino acids and organic acids in GABA-treated creeping bentgrass under drought condition [8].

Recently, we found that exogenous application of GABA enhanced the level of several phytohormones (*trans*-jasmonic acid, salicylic acid, abscisic acid, indole acetic acid, and indole propionic acid) in citrus plants [9]. The gene expression levels of succinic semialdehyde dehydrogenase (SSADH) and GABA-transaminase (GABA-T) were induced in GABA-treated plants seven days post-treatment (dpt), indicating a conversion of GABA to succinate [9]. Succinic dehydrogenase and malate dehydrogenase were also upregulated in GABA-treated plants, indicating an induction of the TCA cycle [9]. The GC–MS analysis showed that the level of endogenous GABA was significantly increased in GABA-treated plants seven dpt, but declined to its normal level 14 dpt, demonstrating that GABA was translocated to the citrus leaves and then was catabolized there [9]. In current study, we used deuterium-labeled GABA (D_6_-GABA) coupled with GC–MS to investigate the translocation, distribution, and metabolism of exogenous GABA in citrus seedlings. The methyl chloroformate (MCF) derivatization used in this study allows quantification of other amino and organic acids. However, for the purpose of our study, we only focused on GABA and its metabolite (succinic acid).

## Results

### Derivatization of GABA standards

**Fig. 1:**
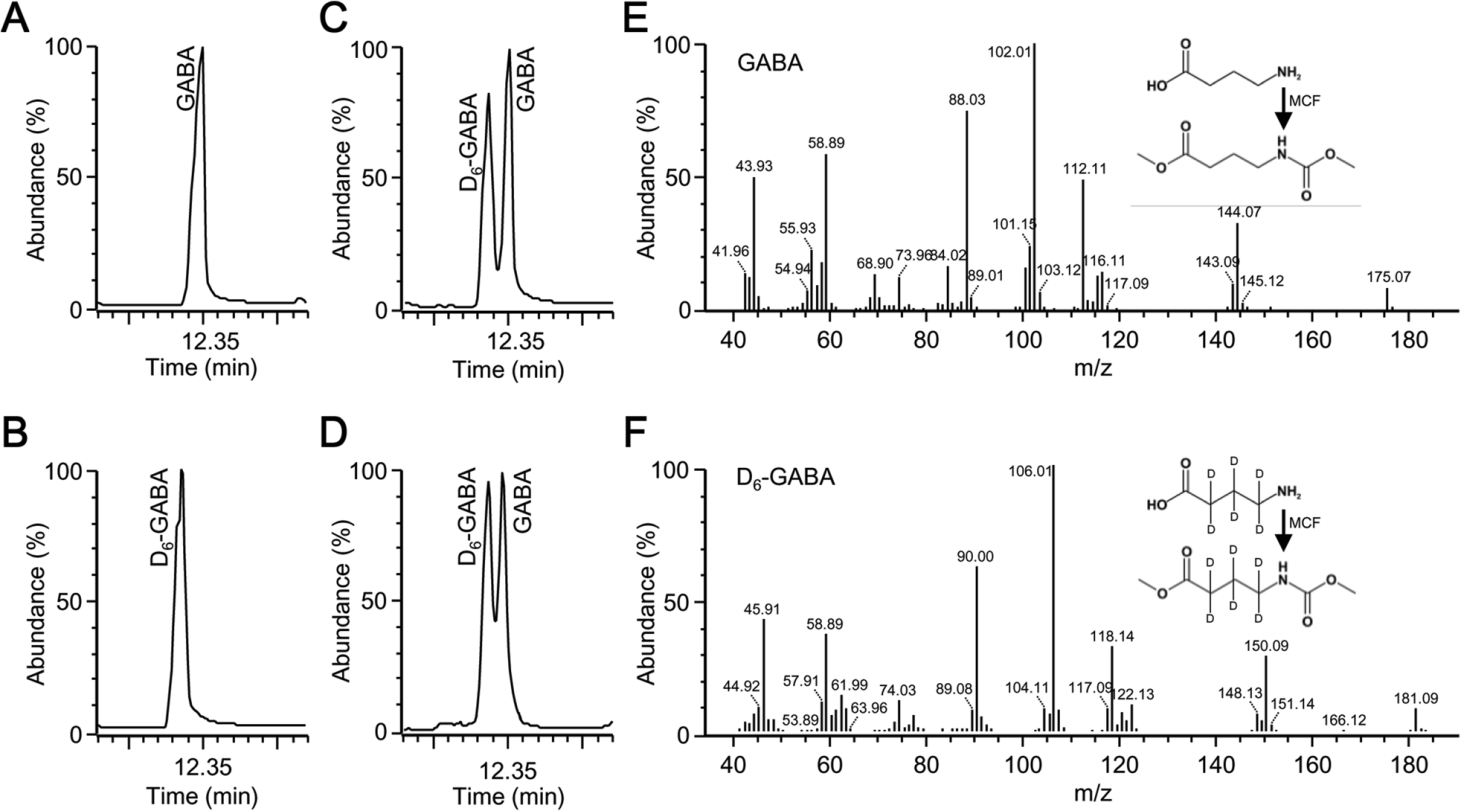
GC–MS chromatograms of the MCF derivatives of GABA and D_6_-GABA and their mass spectra. The MCF derivative of GABA (**a**), D_6_-GABA (**b**), a mixture of GABA and D_6_-GABA (**c**), D_6_-GABA-spiked leaf sample (**d**), and the mass spectra of the MCF derivative of GABA (**e**), and D_6_-GABA (**f**). The reaction scheme of GABA and D_6_-GABA with MCF is also shown in the upper right corner of graph **e, f**

Deuterated (D_6_-GABA) and non-labeled GABA standards were first derivatized separately (Fig. 1a, b) to identify their retention time and mass spectra, and then were mixed and derivatized together (Fig. 1c). The MCF derivative of D_6_-GABA standard eluted before that of the non-labeled GABA (Fig. 1c). The peak of the MCF derivative of D_6_-GABA standard was almost separated from the non-labeled GABA standard (Fig. 1c). In addition, we were able to differentiate between endogenous GABA and exogenous D_6_-GABA (Fig. 1d). The parent ion of the derivatized GABA was 175 (*m/z*) (Fig. 1e), whereas the parent ion of the D_6_-GABA standard was 181 (*m/z*), indicating the presence of six deuterium atoms (Fig. 1f). The mass spectrum of the MCF derivative of GABA showed several main fragments [88, 102, 112, 116, and 144 (*m/z*)] (Fig. 1e), whereas these fragments were shifted in D_6_-GABA to [90], [106], [118], [122], and [150] (*m/z*), respectively (Fig. 1f). These results indicated that GC–MS could easily differentiate between exogenous (D_6_-GABA) and endogenous GABA.

### Percentage recovery of labeled D_6_-GABA

Our GC–MS analysis showed high recovery of the D_6_-GABA (92.6% ± 2.7). No D_6_-GABA was detected in control samples (blank). The level of endogenous GABA in control sample was 84.4 ± 9.8 μg/g. This result showed that D_6_-GABA could be successfully used to study the translocation of GABA in citrus plants. In our preliminary work with detached leaves, we were able to detect D_6_-GABA after 2 h incubation in 10 mM solution. Our preliminary trials also showed that the level of D_6_-GABA was higher (294.1 ± 18.1 μg/g) than endogenous GABA after 6 h incubation in 10 mM D_6_-GABA. This result indicated that GABA was translocated in plants and suggested that 6 h incubation would be sufficient to detect D_6_-GABA in intact plants using GC–MS running in full scan mode.

### Translocation of D_6_-GABA in intact seedlings

**Fig. 2:**
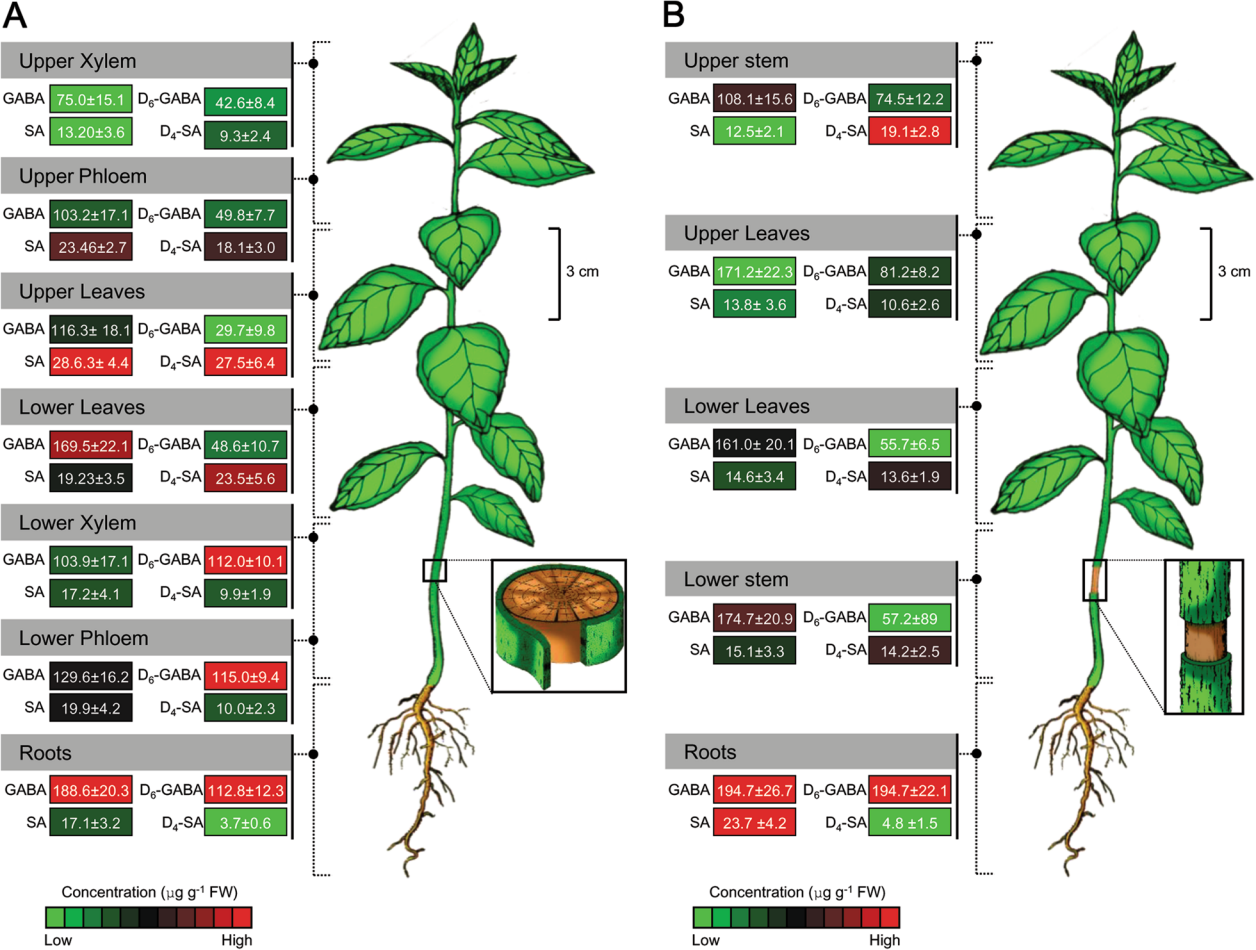
The distribution of endogenous GABA and succinic acid (SA), and exogenous D_6_-GABA and D_4_-SA in non-girdled (**a**) and girdled (**b**) Mexican lime seedling after incubation in 10 mM D_6_-GABA for 6 h. the cortex was referred phloem and the inner stem as xylem

The GC–MS analysis showed that D_6_-GABA was present in the roots, stem and leaves of D_6_-GABA-treated plants (Fig. 2a, b). The concentrations of D_6_-GABA in the treated seedling (non-girdled) at the end of incubation time (6 h) ranged from 28–115 µg/g (Fig. 2a). The concentrations of endogenous GABA in D_6_-GABA-treated plant tissues ranged from 75 to 189 μg/g (Fig. 2a).

**Fig. 3:**
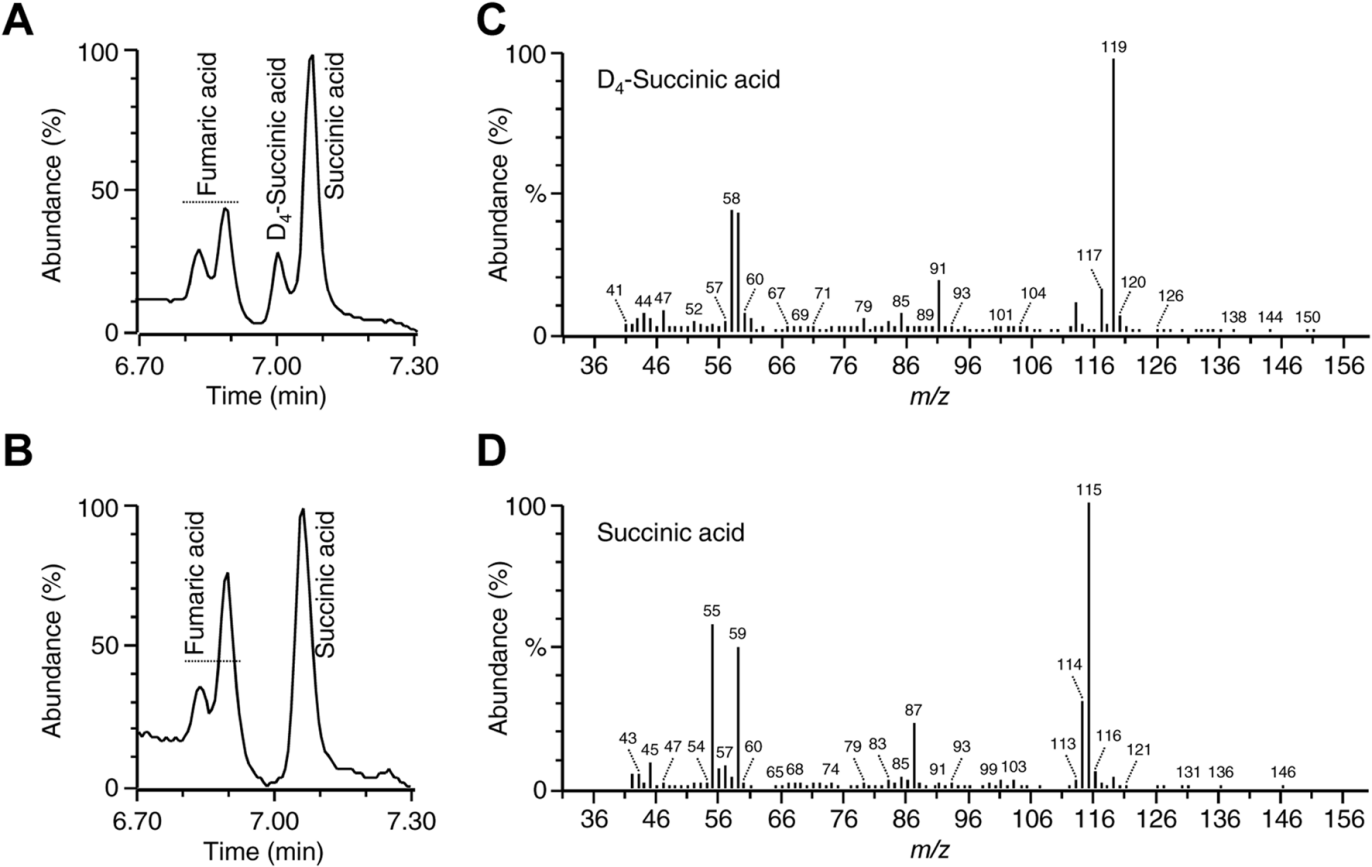
The presence of D_4_-SA in D_6_-GABA-treated plants. A GC–MS chromatogram of the D_6_-GABA-treated plants (**a**) showing the presence of D_4_-SA and the absence of D_4_-SA in control plants (**b**) after derivatization with MCF. The mass spectra of the MCF derivatives of D_4_-SA and SA in D_6_-GABA-treated (**c**) and control plants (**d**)

The GC–MS chromatogram of the D_6_-GABA-treated seedlings also showed a peak at 0.06 min before the succinic acid (Fig. 3a). This peak was absent in the control (Fig. 3b). The retention time of this peak suggested that it could be a D_4_ labeled succinic acid. The mass spectra of this peak showed a base peak of (*m/z*) 119 indicating the loss of a methoxy group and a molecular ion of 150 (*m/z*) (Fig. 3c). Whereas, the mass spectra of the succinic acid peak showed a base peak of 115 (*m/z*) and a parent ion of 146 (*m/z*) (Fig. 3d). The mass spectra result suggested that this peak was a D_4_-labled succinic acid. The 119 (*m/z*) fragment indicated a loss of a methoxy group and the 150 (*m/z*) represented the molecular weight. The concentrations of D_4_-SA in D_6_-GABA-treated seedlings ranged from 4–28 µg/g (Fig. 2a). The level of endogenous succinic acid in D_6_-GABA-treated plants was similar to that of D_4_-SA (Fig. 2a). No D_6_-GABA was detected in treated seedlings that were returned to their original pots 24 h after treatment and only trace amount (1.5 ± 1.8 µg/g) of D_4_-SA was detected in these plants. This result indicated that D_6_-GABA was completely metabolized in treated plants 24 h after treatment.

### Translocation through the xylem; girdling

To test the translocation of D_6_-GABA through the xylem, the main stem was girdled at about 3 cm above the soil surface (Fig. 2b). The D_6_-GABA was detected in the stem and leaves above and below the girdling (Fig. 2b). The concentrations of D_6_-GABA in the different tissues of treated seedling ranged from 56–195 µg/g (Fig. 2b). The concentrations of endogenous GABA in D_6_-GABA-treated plant tissues ranged from 108 to 195 μg/g (Fig. 2b). The presence of D_6_-GABA above the girdled site showed that D_6_-GABA was translocated via the xylem. In the same manner, D_4_-SA was detected in the stem tissues and leaves above and below the girdling. (Fig. 2b) The concentrations of D_4_-SA in the D_6_-GABA-treated seedling ranged from 5–19 µg/g (Fig. 2b). The level of endogenous succinic acid in D_6_-GABA-treated plants was similar to that of D_4_-SA. The level of endogenous succinic acid in D_6_-GABA-treated plants was similar to that of D_4_-SA (Fig. 2b).

### Catabolism of D_6_-GABA in detached leaf

**Fig. 4:**
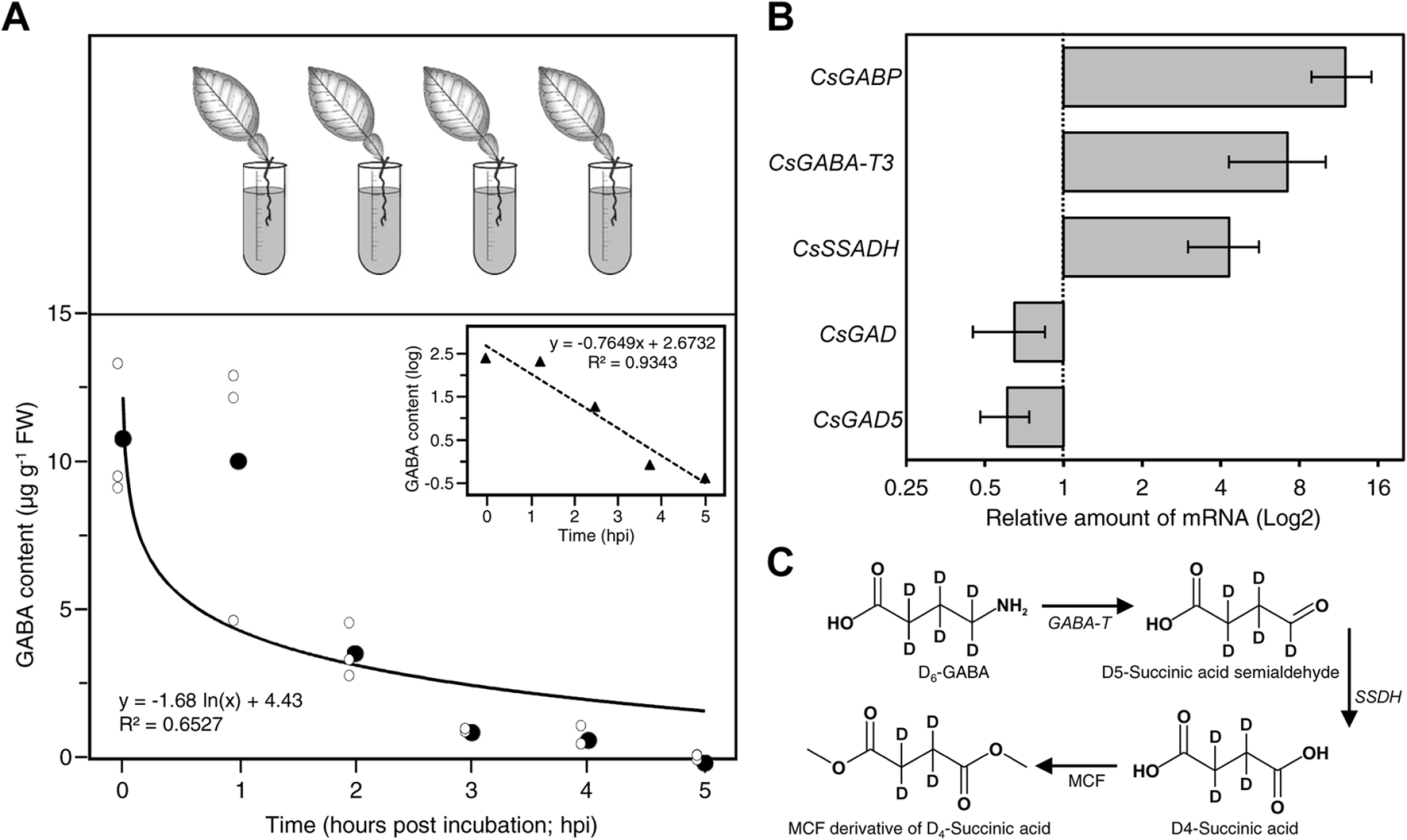
Metabolism of exogenous GABA in plants. The decay (metabolism) of D_6_-GABA in citrus leaves presented in concentration and log of the concentration versus time (**a**). Leaves were incubated in 10 mM D_6_-GABA for 3 h, washed with water, and then transferred to distilled water for 0, 1, 2, 3, 4, and 5 h. Effect of the exogenous D_6_-GABA application on the relative gene expression of several citrus genes involved in GABA biosynthesis and catabolism (**b**). Leaf samples, for RNA extraction, were collected at 6 h post treatment (dpt) with or without GABA [mock (0 mM vs.10 mM GABA]. Bars represents the relative increase in the gene expression level in GABA-treated plants relative to the control, while the error bars represent standard deviation (SDs). Gene expressions were normalized using five housekeeping genes including; elongation factor 1-alpha (*EF1*), F-box/kelch-repeat protein (*F-box*), glyceraldehyde-3-phosphate dehydrogenase GAPC1, cytosolic (*GAPC1,* also known as *GAPDH*), and SAND family protein (*SAND*), which previously showed high stability for transcript normalization in citrus under biotic stress [19–21]. The changes in the gene expression levels were analyzed with the 2^−ΔΔ*C*^_T_ method. Samples were analyzed in triplicate for each biological replicate (*n* = 5). The full list of expressed genes, names, accession numbers, and primers are available in Additional file 1: Table S1. Schematic diagram of the metabolism of exogenous D_6_-GABA in citrus plants. Metabolism of D_6_-GABA to D_4_-succinic acid and derivatization of D_4_-succinic with methyl chloroformate (**c**)

To study the rate of catabolism of D_6_-GABA in citrus leaves, detached leaves were incubated for 3 h in 10 mM GABA solution, washed with distilled water, and then incubated in distilled water for 0, 1, 2, and 4, 5, and 24 h. At the end of incubation time, leaves were analyzed by GC–MS to measure the level of D_6_-GABA. The level of D_6_-GABA in the leaves upon incubation in distilled water was as follows: 0 h; 10.8 ± 2.3 µg/g, 1 h; 10.0 ± 4.6 µg/g, 2 h; 3.7 ± 0.9 µg/g, 3 h; 1.0 ± 0.1 µg/g, 4 h; 0.8 ± 0.4 µg/g, 5 h; not detected (Fig. 4a). The Tukey’s test showed that the level of D_6_-GABA after 2, 3, 4, and 5 h was significantly lower than detected at 0 h. The level of endogenous GABA in D_6_-GABA-treated and control plants ranged from 81.4 to 98.2 µg/g and did not show any significant changes. The level of D_4_-succinic acid in the leaves upon incubation in distilled water was as follows: 0 h; 5.4 ± 1.1 µg/g, 1 h; 3.5 ± 1.67 µg/g, 2 h; 13.1 ± 7.2 µg/g, 3 h; 8.8 ± 2.9 µg/g, 4 h; 7.8 ± 4.6 µg/g, 24 h; 2.0 ± 1.1 µg/g.

### Gene expression

Because there were no significant differences in gene expression between upper and lower leaves, results were pooled and presented together in the heatmap (Fig. 4b).The gene expressions of *gamma*-aminobutyric acid-permease (*GABP*), gamma-aminobutyric acid-transaminase (*GABA-T*) and succinic semialdehyde dehydrogenase (*SSADH*) were upregulated in D_6_-GABA-treated plants (Fig. 4b). The upregulation of the previous genes indicated that exogenous GABA was metabolized to succinic acid. On the other hand, the gene expression of GAD genes was downregulated in D_6_-GABA-treated plants (Fig. 4b), indicating a downregulation of GABA biosynthesis. The GC–MS and the gene expression results together showed that exogenous GABA was metabolized to succinic acid (Fig. 4c).

## Discussion

In our recent work, we showed that the level of endogenous GABA in the leaves of GABA-treated plants was significantly induced seven dpt, and then dropped to its normal level at 14 dpt [9]. This result indicated that GABA was translocated and metabolized in citrus plants. Unfortunately, we did not measure the levels of GABA in other plant parts, such as the roots and stem.

In this study, we used D_6_-GABA to study the translocation and metabolism of exogenous GABA in citrus plants. All the hydrogen atoms attached to carbon are replaced by deuterium in D_6_-GABA. Consequently, this prevents the rearrangement of the deuterium atoms and formation of isotopolog. In addition, our GC–MS results showed that the D_6_-GABA can be easily differentiated from that of the non-labeled GABA. Consequently, using D_6_-GABA we were able to differentiate between the endogenous (non-labeled) and the exogenous (D_6_-labled GABA). The deuterated GABA standards eluted about 0.07 min before the non-deuterated GABA standard. The decrease in the retention time of the deuterated GABA standards could result from the strong bond between the deuterium and carbon atoms [10]. The strong interaction between the deuterium and carbon atoms could decrease the column affinity to D_6_-GABA and consequently results in earlier elution time. Furthermore, the mass spectrum of the D_6_-GABA was different from that of non-labeled GABA; the main fragments in D_6_-GABA were shifted two to six atomic mass unit (amu) compared to those of non-labeled GABA. Analysis of spiked citrus tissues showed high recovery (92.6% ± 2.7) of D_6_-GABA, indicating that D_6_-GABA could be successfully used to study the translocation of GABA in plants.

Incubation of citrus seedling’s roots in D_6_-labled GABA showed that it was translocated from the roots to other tissues within a few hours. D_6_-labled GABA was also detected at high levels in leaves and stem above the girdle, indicating that GABA was transported via the xylem. In addition, D_6_-labled GABA was detected in the cortex and inner stem of non-girdled plants. These results indicated GABA exchange could occur from xylem to phloem and vice versa during long-distance transport. In general, exogenous application of GABA results in an increase in GABA in treated plants. For example, exogenous application of GABA increased the level of endogenous GABA in creeping bentgrass (*Agrostis stolonifera*) under heat stress condition [7, 8]. An increase in endogenous GABA was also reported in several plants including *Caragana intermedia*, *Stellaria longipes*, maize, and sunflower upon exogenous application of GABA [5, 11–13]. The previous results indicated that GABA is translocated in plants.

In agreement with our results, previous studies showed that amino acids can be taken directly by the roots and can be transported between different organs through both xylem and phloem [14]. Plants possess several amino acids transporter families including the GABA-permease (GABP) related family [14]. In our previous study, we showed that citrus genome possesses a putative GABA-permease, which connects the GABA-shunt with TCA cycle by transporting cytosolic GABA to the mitochondria [15]. Interestingly, the gene expression of *GABP* was highly induced in *C*Las-infected and *D. citri*-infested citrus plants, indicating an increase in GABA transport from the cytosol to the mitochondria [15]. In agreement with the gene expression results, the level of succinic acid was also enhanced in *C*Las-infected and *D. citri*-infested plants, indicating a conversion of GABA to succinic acid [15].

The GC–MS results showed that D_6_-GABA was metabolized to D_4_-succinic acid. The upregulation of the gene expression of *GABP, GABA-T*, and *SSADH* supported the GC–MS results. In agreement with our current results, the level of endogenous GABA in citrus plants was increased upon treatment with exogenous GABA seven dpt; however, its level declined to its normal level in few days, suggesting that GABA was translocated and metabolized [9]. This result was supported by the increased gene expression of GABA-transaminase (*GABA-T*) and succinic semialdehyde dehydrogenase (*SSADH*) in GABA-treated citrus plants seven dpt [9]. In addition, the gene expressions of malate dehydrogenase and succinic dehydrogenase genes were highly induced in GABA-treated plants seven dpt, indicating that GABA was metabolized to succinate and fed into the TCA [9].

The half-life of D_6_-GABA in citrus leaves was about 1.3 h, indicating that GABA is quickly converted to succinic acid. In addition, no D_6_-GABA was detected in seedlings 24 h upon incubation for 6 h in 10 mM D_6_-GABA, confirming that it was completely metabolized to succinic acid. Recently, we showed that the levels of GABA and succinic acid were significantly increased in detached citrus leaf after 1 h incubation in 10 mM GABA, indicating that GABA was quickly metabolized to succinic acid [16]. In addition, the level of fumaric acid was significantly increased in citrus leaves after 1 h incubation in 10 mM GABA, indicating that succinic acid was fed into the TCA cycle [16]. Unfortunately, no D_2_-labeled fumaric acid was detected in D_6_-GABA-treated plants in this study. The absence of D_2_-labeled fumaric acid in D_6_-GABA–treated plants could result from the exchange of deuterium atoms in deuterium-labeled succinic acid with hydrogen atoms by succinic acid dehydrogenase. An exchange of deuterium atoms with hydrogen atoms of the solution was reported in in deuterium-labeled succinic acid in the presence of succinic acid dehydrogenase, which catalyzes the oxidation of succinic acid to fumaric acid [17]. Previous studies suggested that succinic semialdehyde could also be reduced to gamma-hydroxybutyrate (GHB) in plants under a variety of abiotic stresses [18]. However, we could not check for GHB because it cannot not be derivatized using methyl chloroformate. Therefore, a future study with a different detection or derivatization method is needed to test for the formation of GHB.

## Conclusions

Our results showed that exogenous GABA was taken up by the roots and was transported via the xylem to the leaves and other plant tissues in the upper part of the plant. In addition, our results showed that GABA was quickly metabolized to succinic acid. Our results also demonstrated that D_6_-GABA could be successfully used to distinguish between endogenous and exogenous GABA in plants. Furthermore, our result suggested that isotope-labeled GABA could be a valuable tool to study the translocation and metabolism of this important signaling molecule in plants.

## Methods

### Plant materials

Mexican lime (*Citrus aurantifolia*) was used in this study. Seeds were potted in plastic cones (20 × 4 cm) containing Sungro professional growing mix (Sungro Horticulture, Agawam, MA). Seedlings were kept in a greenhouse (28 ± 1 °C, 60 ± 5% relative humidity, L16:D8 h photoperiod) at the Citrus Research and Education Center (CREC), University of Florida, Lake Alfred, Florida. Seedlings were watered twice weekly. At the time of experiment, plants were about three-month old and around 15 ± 5 cm tall.

### Preparation of GABA standards

D_6_-GABA and GABA standards were purchased from Sigma Aldrich (St. Louis, MO). Stock solutions of GABA (10 mM), D_6_-GABA (10 mM), and succinic acid (10 mM) were prepared daily using distilled water. A set of serial dilutions were made and used to construct the standard curves.

### Percentage recovery of D_6_-GABA

Citrus leaves were ground with liquid nitrogen using a mortar and pestle and 100 mg of the ground tissue was spiked with 10 µl of D_6_-GABA (10 mM). Five samples were spiked with D_6_-GABA standard and five controls were mixed with 10 µl of distilled water, then were extracted and analyzed as outlined below to determine the percentage recovery. Percentage recovery was calculated by dividing the area of the D_6_-GABA peak in spiked sample by the area obtained from D_6_-GABA standard (10 µl of 10 mM).

### Preliminary work: intake of D_6_-GABA by a citrus leaf

To study the uptake of D_6_-GABA by a single citrus leaf, the petiole was cut, under water, using a sharp blade and was quickly immersed in 10 mM D_6_-GABA solution. Incubation was carried out during daytime inside a greenhouse at the same condition described above. At the end of the incubation time (1, 2, 4, and 6 h), the petiole was cut and discarded, and the leaf was washed for 1 min with distilled water to remove any adsorbed D_6_-GABA from the leaf surface.

### Treatment of citrus plants with D_6_-GABA

Before treatment with D_6_-GABA, citrus plants were removed from their original pots and the roots were washed with distilled to remove the soil. To determine if GABA was transported in the xylem, the main stem of five plants was completely girdled before incubation. Girdling was done by complete removal of a 1 cm wide strip of bark about 3 cm above the soil surface (Fig. 2). Then 15 plants (5 girdled and 10 non-girdled) were incubated in 10 mM D_6_-GABA for 6 h. Each plant was placed in a 5 ml plastic centrifuge tube and the roots were covered with 10 mM D_6_-GABA solution. Control plants (5 plants) were incubated in distilled water. Incubation was carried during daytime inside a greenhouse at the same condition described above. At the end of incubation time (6 h), plants were washed for 1 min with distilled to remove any adsorbed D_6_-GABA. Five of the D_6_-GABA-treated plants were returned to their original pots (potting mix) and were analyzed after 24 h. The rest of the plants were dissected and analyzed by GC–MS. For these analyses, the stem bark was dissected into the cortex and the inner stem (xylem). Three leaves from the upper part of each plant (mature, moderate-age, and juvenile), were collected and pooled together (upper leaves). In the same manner, three leaves from the lower part of each plant (mature, moderate-age, and juvenile), were collected and pooled together (lower leaves).

### Catabolism of D_6_-GABA by citrus leaf

To study the catabolism rate of GABA in citrus leaves, the leaf petioles were immersed in 10 mM D_6_-GABA for 3 h. At the end of incubation time, the leaves (30 leaves) were removed from the D_6_-GABA solution and washed with distilled water. Then, sets of five leaves were kept in distilled water for 0 h, 1 h, 2 h, 3 h, 4 h, and 5 h, respectively. Incubation was carried during daytime inside a greenhouse at the same condition described above.

### Analysis of GABA using GC–MS

GABA was extracted from ground tissues (0.10 ± 0.002 g) using methanol 80% containing 0.1% HCl 6 N according to the procedures described in our previous study [9]. Briefly, plant tissues were ground with liquid nitrogen using a mortar and pestle. Then, 100 mg was transferred to 2 ml centrifuge tube and 700 µl of the solvent mix was added and the sample was vortexed for 30 s. The sample was kept for 10 min in ice and then was centrifuged for 10 min at 17,000×*g* at 5 °C. The supernatant was decanted to a new centrifuge tube and the extraction procedure was repeated two more times by adding 700 µl of the solvent mix each time. The collected extract was evaporated under a gentle nitrogen stream and was re-dissolved in 200 µl of the solvent mix, centrifuged again, and transferred to a silanized conical 1-ml insert. The extract was concentrated to about 40 µl under nitrogen stream. The extract was derivatized with methyl chloroformate (MCF) as described in our previous study [9]. Briefly, 180 µl of 1 N sodium hydroxide, 167 µl of methanol, and 34 µl of pyridine was added to the sample extract or standard and the mixture was vortexed for 10 s. Then, 20 µl of MCF was added and the mixture was vortexed for 30 s. Another 20 µl of MCF was added and the mixture was vortexed again for 10 s. A 100-µl aliquot of chloroform was added and the mixture was vortexed for 10 s. Finally, 200 µl of 50 mM sodium bicarbonate was added and the mixture was vortexed for another 10 s. The chloroform layer (bottom) was transferred to a silanized GC–MS insert and 5 mg of sodium sulfate were added.

For GC–MS analysis, 0.5 µl of the derivatized sample was injected into the GC–MS running in the full scan mode. Derivatized samples and standards were analyzed using a Clarus 680 gas chromatograph equipped with Clarus SQ 8 T mass spectrometer running in the electron ionization mode (EI) (Perkin Elmer, Waltham, MA, USA). The system was fitted with a ZB-5MS GC column (5% Phenyl-Arylene 95% Dimethylpolysiloxane; low bleed, 30 m × 0.25 mm × 0.25 µm film thickness; Phenomenex, Torrance, CA, USA). The flow rate of the helium carrier gas was set at 0.9 ml/min. The GC thermo-program was as follows: initial temperature was held at 70 °C for 4 min, and then increased to 280 °C at a rate of 10 °C/min, and finally held for 5 min. The injector was set at 220 °C, inlet line at 200 °C, the source temperature at 180 °C, and the electron energy was set to 70 eV. The GC–MS chromatograms analysis were performed as described in our previous study [9]. Calibration curves were constructed from the linear regressions obtained by plotting the concentration vs. peak area for each standard. The level of endogenous succinic acid and exogenous (D_4_-succinic acid) was quantified using succinic acid standard curve.

### Gene expression analysis using quantitative real time PCR (RT-PCR)

Leaf samples, for RNA extraction, were collected at 6 h post treatment (dpt) with or without GABA [mock (0 mM vs.10 mM GABA]. Leaves were sampled from the top and the bottom as described above. The RNA was extracted from (0.1 ± 0.002 g) ground leaf tissues using TriZol® reagent (Ambion®, Life Technologies, NY, USA). The quantity and quality of isolated RNA were assessed using NanoDrop 2000 spectrophotometer (Thermo Scientific, Waltham, MA, USA). The cDNA primers were synthesized using SuperScript first-strand synthesis system (Invitrogen, Waltham, MA, USA) according to the manufacturer’s instructions. The qPCR was performed on an ABI 7500 Fast-Time PCR System (Applied Biosystems, Waltham, MA, USA) using SYBR Green PCR master mix (Applied Biosystems). Samples were analyzed in triplicate for each biological replicate for each treatment. Primers for genes involved in the GABA shunt pathway were used to measure the gene expression (Additional file 1: Table S1). The relative expression of the consensus sequence among PCR products was done according to the 2^−ΔΔ*C*^_T_ method [18]. Four genes were used as endogenous genes (reference genes) to normalize the data of gene expression including; elongation factor 1-alpha (*EF1*), F-box/kelch-repeat protein (*F-box*), glyceraldehyde-3-phosphate dehydrogenase GAPC1, cytosolic (*GAPC1,* also known as *GAPDH*), and SAND family protein (*SAND*) [19–22].

### Statistical analysis

Data were analyzed using JMP 9.0 software (SAS, Cary, NC). Analysis of variance (ANOVA) followed by post hoc pairwise comparisons using Tukey–Kramer honestly significant different test (Tukey HSD) were used to compare level of D_6_-GABA among the different treatments (*p* < 0.05).

## Supplementary information

**Additional file 1: Table S1. **Primer used for gene expression analysis of GABA shunt-associated enzymes by real time RT-PCR.

## Data Availability

Datasets used for the study can be requested from the corresponding author on reasonable request.
