# Supplementary material for: The use of deuterium-labeled gamma-aminobutyric (D6-GABA) to study uptake, translocation, and metabolism of exogenous GABA in plants
Source: Plant Methods. 2020 Feb 27;16:24. doi: 10.1186/s13007-020-00574-9 (PMC7045438; doi:10.1186/s13007-020-00574-9)
Supplement: Supplementary file 1 — Additional file 1: Table S1. Primer used for gene expression analysis of GABA shunt-associated enzymes by real time RT-PCR. [file 13007_2020_574_MOESM1_ESM.docx]

**Table S1.** Primer used for gene expression analysis of GABA shunt-associated enzymes by real time RT-PCR^a^.

| **Gene** | **Accession ID** |  | **Primer**  **(Forward and Reverse)** | **TM**  **(˚C)** | **Product size**  **(bp)** |
| --- | --- | --- | --- | --- | --- |
| ***CsGABP*** | XM_006468698.3 | F | TTCATCCCCGGACCTTTCAA | 58.93 | 197 |
|  |  | R | CGAGCACTGAAGATCCAAGC | 58.99 |  |
| ***CsGABA*-*T3*** | XM_006481305.2 | F | CTCCTGAATGGGGGATAGGT | 60.15 | 196 |
|  |  | R | TGCTGGGACTTGAGTTCCTT | 59.84 |  |
| ***CsGAD*** | NM_001288909.1 | F | ATAGCCCGACTGTTCAATGC | 60.10 | 199 |
|  |  | R | ATTTCTCCCAGCAGACCTGA | 59.80 |  |
| ***CsGAD5*** | XM_006478039.2 | F | CCTTGACGGGAGAATTTGAG | 59.66 | 200 |
|  |  | R | GCCGTACTTGTGACCACTGA | 59.75 |  |
| ***CsSSADH*** | XM_006493686.2 | F | CAACGTACATGGCGTGTCTC | 60.18 | 200 |
|  |  | R | CTGGCCCATCATTTTCTGTT | 59.93 |  |
| **Reference genes** | | | | | |
| ***CsEF1* ^b^** | AY498567.1 | F | GGAAGTTCGAGACCACCAAG | 59.70 | 202 |
|  |  | R | ACACCAAGGGTGAAAGCAAG | 60.15 |  |
| ***CsF-box* ^b^** | XM_006482390.1 | F | ACTTGACAGATGGGCTGTCC | 60.12 | 197 |
|  |  | R | CAGCAACCAAATACCCGTCT | 59.99 |  |
| ***CsGAPC1* ^b^** | XM_006483974.2 | F | ACTCCAGAGGGATGATGTGG | 59.92 | 200 |
|  |  | R | ATGGGATCTCCTCTGGGTTC | 60.28 |  |
| ***CsSAND* ^b^** | XM_006488024.2 | F | GCATCAGCTGCACAGAAGAG | 59.89 | 204 |
|  |  | R | GGAATGTAGCTGGGTTCCAA | 59.93 |  |

**^a^** The listed genes were assembled based on recent available data in GenBank, National Center for Biotechnology Information website (NCBI, <http://www.ncbi.nlm.nih.gov/gene/>).

**^b^** Genes have been used as a reference/housekeeping genes for data normalization according to [19–21].

**Abbreviations**

***CsGABP:*** GABA permease (aka amino-acid permease BAT1-like isoform X1 [*C. sinensis*])

***CsGABA*-*T3***: PREDICTED: *Citrus sinensis* gamma aminobutyrate transaminase 3

# *CsGAD*: *Citrus sinensis* glutamate decarboxylase-like

# *CsGAD5*: PREDICTED: *Citrus sinensis* glutamate decarboxylase 5-like

# *CsSSADH*: PREDICTED: *Citrus sinensis* succinate-semialdehyde dehydrogenase, mitochondrial

***CsEF-1α***: *Citrus sinensis* elongation factor-1 alpha

***CsF-box****:* *Citrus sinensis* F-box/kelch-repeat protein

***CsGAPC1****: Citrus sinensis* glyceraldehyde-3-phosphate dehydrogenase, cytosolic (aka GAPDH)

# *CsSAND*: *Citrus sinensis* SAND family protein
